# A magical experience or a puzzle: how do people watch conjuring tricks?

**DOI:** 10.7717/peerj.21742

**Published:** 2026-09-25

**Authors:** Dawson von Stein, Richard Wiseman, David C. Watson, Rodney Schmaltz

**Affiliations:** 1 Department of Psychology, MacEwan University, Edmonton, Alberta, Canada; 2 Department of Psychology, University of Hertfordshire, Hatfield, Hertfordshire, United Kingdom

**Keywords:** Magic, Psychometrics, Wonder, Awe, Paranormal, Belief, Rational, Intuition, Conjuring, Perception

## Abstract

Conjuring tricks often contain two components: The effect (an apparently impossible happening) and the method (the secret of the trick). Prior scales have assessed individuals’ general liking for magic, but not examined their interest in the effect and method. This paper validates a new scale (Method-Effect Scale; MES) measuring people’s preference for these two components and examines its relationship with several variables including a general liking for magic, wonder, pseudoscientific beliefs and thinking style. Adult participants (*N* = 922) completed an online survey containing the MES, Loathing of Legerdemain Scale, Capacity for Wonder Scale (CFW), Inventory of Epistemically Unwarranted Beliefs Scale (EUB), Rational Experiential Inventory (REI), and demographic questions. An exploratory factor analysis revealed that the MES had two distinct factors (Effect and Method) and strong internal reliability (overall *α* = .83: method *α* = .86: effect *α* = .77). Significant positive correlations emerged between both MES subscales, the CFW subscales, and the EUB scientific knowledge subscale. The MES Method subscale was positively correlated with the REI’s Need for Cognition and Faith in Intuition subscales, and negatively associated with some EUB subscales (conspiracy theory, religion, and health). The MES Effect subscale was positively associated with the EUB ghost subscale and negatively correlated with the conspiracy theory subscale. These results suggest that the MES is a valid and reliable measure, and we discuss how this new measure can help researchers obtain a more fine-grained insight into participant’s perception of magic, and how magicians can employ it to improve their performances.

## Introduction

Academic research into the psychology of conjuring has a surprisingly long history that dates to the late 1800s (*e.g.*, Binet, 1894; Jastrow, 1896). The last few decades have proved especially productive (*e.g.*, Lamont & Wiseman, 1999; Schiffman, 2005; Macknik, Martinez-Conde & Blakeslee, 2010; Kuhn, 2019), with researchers investigating a range of topics, including the psychology employed by magicians to fool their audiences (*e.g.*, Kuhn, Amlani & Rensink, 2008), how witnessing magic tricks impacts paranormal belief (*e.g.*, Mohr, Koutrakis & Kuhn, 2015), and whether learning magic tricks boosts mental and physical wellbeing (*e.g.*, Wiseman, 2023).

As part of this work, Silvia et al. (2023) adopted an individual differences approach to examine why people tend to like or dislike magic. The researchers developed the Loathing of Legerdemain Scale (LOLS) and then used it to explore three hypotheses. First, the notion that people with a tendency to experience curiosity and awe would find magic more enjoyable was supported by significant negative correlations between participants’ scores on the LOLS, the Openness to Experience sub-scale of the Big Five Inventory-2, and the Absorption sub-scale of the Unusual Aesthetic Experiences Scale. Second, the hypothesis that some people would dislike watching magic because the experience generated a sense of uncertainty was supported by significant positive correlations between participants’ scores on the LOLS, the Brief Dogmatism Scale and the Intolerance of Uncertainty Scale. Finally, the idea that some people would be attracted to magic because it represents a form of interpersonal manipulation was supported by strong negative correlations between participants’ scores on the LOLS and the Agreeableness sub-scale of the Big Five Inventory-2, and significant positive correlations between scores on the LOLS, the Dominance sub-scale of the Dominance-Prestige Scale, and the psychopathy and sadism sub-scales of the Dirty Dozen Scale. The current study builds on this work by taking a more fine-grained look at the perception of magic.

Most texts about the theory of magic note that conjuring tricks consist of two key elements: an effect and a method (*e.g.*, Lamont & Wiseman, 1999; Rensink & Kuhn, 2015). The effect is an apparently impossible happening (*e.g.*, the magician shows an empty box and then produces some flowers from the box) whilst the method is the secret that makes the trick work (*e.g.*, the flowers were hidden in a secret compartment within the box). Additional work in this area is especially timely due to a growing interest in the distinction between methods and effects (*e.g.*, Kuhn, Thomas & Griffiths, 2026).

Spectators seem to express a preference for, and attach greater value to, one of these two components. For instance, qualitative work conducted by Medeiros et al. (2022) involved asking those attending an exhibition on the science of magic what they liked about magic. Some respondents focused on the effect (mentioning the feelings that magic evoked, the sense of believing the impossible, and child-like wonder) whilst others were more method-oriented and eager to try to work out the secrets to tricks. Similarly, in a survey reported by Jay (2016), members of the public were asked what they liked most about magic. Many went with effect-based answers (*e.g.*, being amazed and surprised) whilst others said that they enjoyed trying to figure out the solution to an illusion. In short, some spectators enjoy focusing on the effect and thus value experiencing a seemingly impossible event. To them, the secret behind the trick is less important. In contrast, other spectators are more method-oriented and enjoy trying to figure out how the trick was achieved. To them, magic tricks are puzzles in need of a solution. The fundamental nature of these different perspectives sits at the heart of a clever magic trick (called The Honour System) created by the American illusionists Penn and Teller (see Donnelly & Rosenblatt, 2019). At the start of their illusion, the audience sees Teller being locked inside a large wooden trunk. Next, Penn announces that his partner will escape from the trunk and that each member of the audience can experience this illusion in one of two ways: They can either close their eyes and experience a seemingly impossible escape (the effect) or keep their eyes open and see the secret to the trick (the method). Although previous work tends to frame the choice as binary, it is also plausible that preferences for effects and methods represent two independent dimensions rather than opposite ends of a single continuum. That is, some spectators may score high on both orientations, enjoying the experience of impossibility while also relishing the challenge of figuring out the secret.

Spectators’ preferences for experiencing effects or figuring out methods have yet to be embraced by researchers examining the psychology of conjuring. This is unfortunate as exploring these preferences, and determining whether they function as independent dimensions or opposing orientations, has the potential to make a valuable contribution to several areas of research. For example, psychologists interested in conjuring may obtain more fine-grained insights from grouping participants as effect or method oriented in much of their work, including studies exploring why people like magic and the impact of learning magic on well-being. From a more general perspective, investigating the effect/method dichotomy may also help psychologists concerned with conceptually similar areas, including the perception of optical illusions (Tyler, 2022), research into narrative transportation (Thomas & Grigsby, 2024) and the development of dual-processing models (Zielonka et al., 2024). Finally, exploring this aspect of audience psychology is likely to have practical benefits for magicians and related performers, including helping them craft illusions and presentations that appeal to specific audiences, and developing ways of shifting spectators from one orientation to another. For example, it could be that the two groups perceive optical illusions differently, with more method-oriented individuals being curious about how the illusion works whilst more effect-based observers enjoying the surprising experience. Similarly, when developing magic-based wellness interventions, method-oriented participants might derive more benefit from focusing on the secrets to tricks whilst effect-based participants would respond more to being able to evoke a magical experience in audiences. Finally, performers might change their presentations around the two perspectives, explicitly framing a performance as an experience to be enjoyed rather than a problem to be solved.

The current study involved two parts. In the first, adult participants completed a series of self-report items that formed the basis of a new measure entitled the Method-Effect Scale (MES). The MES was designed to assess method and effect orientations as separate dimensions, allowing individuals to score high or low on either or both. A second part of the work provides an exploratory and initial examination of the relationship between the MES and five areas: magic engagement, the liking of magic, intuitive and rational thinking styles, wonder, and pseudoscientific beliefs. Each of these topics will be discussed in turn.

The first of these five areas concerned participants’ liking for magic, which was measured using the LOLS. It was predicted that the more people enjoy magical effects and searching for methods, the more they will enjoy magic, and thus significant negative correlations were expected between participants’ scores on the MES Effect and Method sub-scales and the LOLS. To obtain further insights into people’s engagement with magic, their LOLS scores were supplemented with a series of quantitative and qualitative questions relating to how often participants watched magic on television/online and/or attended live shows (Engagement Survey; ES). The quantitative data is presented in this paper, and it is predicted that participants’ scores on the MES Effect and Method sub-scales would positively correlate with each of these questions.

Second, participants’ decision-making and thinking preferences were measured *via* the Rational Experiential Inventory 10 (REI-10: Pacini & Epstein, 1999). This psychometrically valid short-form measure is frequently used by psychologists exploring dual-processing and contains two sub-scales: Faith in Intuition and Need for Cognition. Thinking-style effects are strongest when task demands fit the strengths of that style (*e.g.*, Phillips et al., 2016). The MES Effect score was therefore expected to align with intuitive cognition, because attending to an apparent impossibility appears more closely aligned with experiential processing than the analytic work of explaining its method (Pacini & Epstein, 1999; Marks et al., 2008; Bouvet & Bonnefon, 2015; Gronchi & Zemla, 2021). MES Effect scores were predicted to correlate positively with Faith in Intuition and negatively with Need for Cognition.

Third, participants completed the Capacity for Wonder scale (CFW: Geller et al., 2020). Psychologists view wonder as a complex emotion that can be elicited when people encounter stimuli that appear at the edge of their understanding, and therefore prompts a need for revision (De Cruz, 2024). The CFW assesses the disposition to experience this emotion and has been used to examine the role that wonder plays in professional education and lifelong learning (Geller et al., 2020). It has two sub-scales: Perspective shifting (curiosity and an openness to challenging an existing understanding of the world) and emotional reawakening (being drawn to events that elicit surprise and amazement). Research (for a review, see De Cruz, 2024) suggests that wonder can be elicited by experiencing a seemingly remarkable event (*e.g.*, the beauty of a rainbow) or understanding the nature of that event (how rainbows result from the refraction and reflection of sunlight and water droplets). As a result, it was predicted that participant’s scores on both the MES Effect and Method sub-scales would positively correlate with their scores on the two sub-scales of the CFW.

Fourth, participants completed the Inventory of Epistemically Unwarranted Beliefs (Dyer & Hall, 2019; EUB). This measures the degree to which people endorse beliefs that are not supported by scientific evidence, and provides a general score along with seven subscales (Paranormal, Religion, Health, Extraordinary Life Forms, Conspiracy Theories, Ghosts, and Scientific Knowledge). Recent work suggests that the relationship between interest in magic and such beliefs is complex. Silvia et al. (2025a) found that positive attitudes toward magic were associated with two distinct profiles: one characterized by higher paranormal and superstitious beliefs, and another characterized by higher critical thinking and skepticism. Additionally, Silvia et al. (2025b) found that some audience members attribute magicians’ performances to genuine supernatural abilities, and that such attributions were associated with belief in psychic powers and precognition. However, enjoying a magic performance does not require believing that the events are genuinely supernatural; spectators can appreciate the experience of impossibility while remaining fully aware that a trick is involved. Given this complexity, the relationship between the MES subscales and the EUB was treated as exploratory. It was tentatively expected that the MES Method subscale would correlate positively with the EUB Scientific Knowledge subscale, given that method-oriented individuals actively engage in analytical reasoning about how tricks work. No strong directional predictions were made for the remaining EUB subscales. All analyses were exploratory, and all data exclusions and measures have been reported.

## Materials & Methods

### Participants

Undergraduate students (*N* = 1,006: Mean age = 20.36 years, *SD* = 3.67: 38.3% identified as male, 59.9% as female, 1.2% as gender non-conforming, and .6% preferred not to respond) were recruited from a Canadian university and received course credit for participating in the survey. The final sample was created by removing incomplete responses for any participant missing two or more responses and those who failed an attention check (*N* = 922; Mean age = 20.40 years, *SD* = 3.71; 38.6% identified as male, 59.5% as female, 1.2% as gender non-conforming, and .7% preferred not to respond).

### Instruments

#### Method-Effect Scale

The MES is an author-developed scale that measures an individual’s attitudes toward magic by assessing their interest in the method and effect components of magic tricks. The scale consists of 10 items (*e.g.*, “When a magician performs, I love seeing something that should be impossible”), divided into two subscales. The first subscale contains five questions that ask about a participant’s interest in the effect that arises from a magic trick. The second subscale contains questions that ask about interest in the mechanisms behind magic tricks. The scale features a five-point Likert Scale ranging between ‘1’ (strongly disagree) and ‘5’ (strongly agree).

#### Inventory of Epistemically Unwarranted Beliefs (Dyer & Hall, 2019)

The EUB assesses the degree to which respondents endorse beliefs lacking robust empirical support, including those associated with paranormal claims, conspiracy theories, and pseudoscientific topics. It contains 52 items (*e.g.*, There are benevolent spiritual beings who act as protectors or guides for human beings), with respondents indicating the degree to which they endorse each item on a five-point scale between ‘1’ (sure it’s false) and ‘5’ (sure it’s true). The scale contains six epistemically unwarranted belief subscales (Ghosts, Health, Conspiracy Theories, Paranormal, Extraordinary Life Forms, Religion), and one science knowledge subscale, with higher scores indicating a greater belief in each subscale. The EUB has robust internal consistency (Cronbach’s *α* ranging from .59 to .85 across six subscales and an overall *α* of .93 for the EUB total, excluding science knowledge) and principal components analysis supports a stable six-factor structure for the scale in college student samples (Dyer & Hall, 2019).

#### Loathing of Legerdemain Scale (Silvia et al., 2023)

The LOLS measures respondents’ negative attitudes toward magic and illusions. Respondents rate four statements about their attitude towards magic (*e.g.*, ‘I find magic tricks annoying’) on a five-point scale between 1 (Strongly disagree) and 5 (Strongly agree). Higher scores indicated a stronger dislike of magic. The scale was validated using a multinational sample of 1,599 adults and demonstrated strong internal consistency with a Cronbach’s Alpha of *α* = .86 (Silvia et al., 2023). In the current sample, the Cronbach’s alpha for the LOLS was *α* = .86.

#### Capacity for Wonder Scale (Geller et al., 2020)

The CFW assesses respondents’ tendency to experience key cognitive and emotional aspects of wonder, such as drawing new connections, pausing to reflect, experiencing amazement during ordinary events, and feeling surprise or seeing the world with the interest of a child. It contains 10 items (*e.g.*, ‘Indicate how likely you are to find yourself drawing new connections between things in the world’), with respondents indicating the degree to which they endorse each item on a six-point scale between ‘1’ (not at all likely) and ‘6’ (extremely likely). The scale contains two subscales (Perspective Shifting and Emotional Reawakening), with higher scores indicating a greater belief in each subscale. The scale demonstrates strong psychometric properties, including a stable two-factor structure and high internal consistency with a Cronbach’s Alpha of *α* = .87 (Geller et al., 2020). In the current study, the total CFW had a Cronbach’s Alpha of *α* = .83, the perspective shifting subscale had a Cronbach’s Alpha of *α* = .80, and the emotional reawakening subscale had a Cronbach’s Alpha of *α* = .76.

#### Rational–Experiential Inventory-10 (Pacini & Epstein, 1999)

The REI-10 is a brief version of the Rational–Experiential Inventory developed to measure individual differences in analytic (rational) and intuitive (experiential) thinking styles. It contains 10 items (*e.g.*, ‘I prefer complex to simple problems’), with respondents indicating the degree to which they endorse each item on a five-point scale between ‘1’(completely false) and ‘5’ (completely true). The scale contains two sub-scales (Rational, reflecting analytical, logical thinking and Experiential, reflecting intuitive, affect-based processing), with higher scores indicating participants are higher in these traits. The REI-10 demonstrated good internal consistency, with Cronbach’s Alpha of.78 for the Rational subscale and .77 for the Experiential subscale (Pacini & Epstein, 1999). For the current study, the Cronbach’s Alpha was .66 for the rational subscale and .80 for the intuitive subscale.

#### Engagement Survey

This survey was created by the authors and measured engagement with magic-oriented material (*e.g.*, watching magic videos online, following magicians on social media, attempting to perform simple magic tricks). It contains five items (*e.g.*, ‘How often do you watch magic performances online or on television?’), with respondents indicating the degree to which they endorse each item on a five-point scale between ‘1’ and ‘5’. The participants were then asked four open questions about magic (*i.e.,* who is/are your favourite magicians). The five quantitative items were combined into a single measure, with higher scores indicating greater engagement with magic materials. The scale had good reliability, with a Cronbach’s Alpha of .72. All items are included in Appendix A.

Where applicable, the authors have permission to use all of these instruments from the copyright holders.

### Procedure

The MacEwan Research Ethics Board granted approval for the protocol (approval number: 102448). Participants were recruited *via* the MacEwan University student psychology pool through Sona systems and completed the survey online in their own time *via* the Qualtrics platform. After providing written consent, participants were asked demographic questions (*i.e.,* their age, gender, year of study, first language and other languages they fluently speak). Next, the participants completed the author-developed MES, EUB, LOLS, CFW, REI-10, and Engagement Survey. The measures were presented in a fixed order, with the MES administered first so that responses to the new scale were not influenced by the content of the other questionnaires. There was one attention check that asked the participant to select the letter “c”. The time taken for each participant to complete the survey was recorded, but response time was not used as an exclusion criterion. The final sample was created by removing 84 participants who were either missing two or more responses or failed an attention check. After completing the study, participants were debriefed and awarded research credit.

## Results

### Exploratory factor analysis and examination of the Method-Effect Scale

We hypothesized that the MES would contain two factors: one measuring the effect and the other the method component of magic tricks. To assess the validity of the *a priori* conceptualization of this structure, an exploratory factor analysis using oblimin rotation was conducted (Table 1).

**Table 1:** *A priori* factor structure of the MES. The two factors of the MES.

**Hypothesized factor**
**Method (1)**
6. I enjoy trying to figure out how magic tricks were achieved.
7. To me, magic tricks are puzzles to be solved.
8. I enjoy thinking about how magic tricks might have been done, even if I can’t find solutions.
9. I like to watch magicians carefully to see if I can catch them out.
10. I love finding out the secrets to magic tricks.
**Effect (2)**
1. When a magician performs, I love seeing something that should be impossible.
2. I enjoy the feeling of astonishment and wonder at a magic show.
3. I like suspending my disbelief when the magic seems to happen.
4. When I see a magic show, it feels like I have entered a world where anything is possible.
5. I am not really interested in finding out the secret to a magic trick.

The results of the exploratory factor analysis are presented in Table 2. Nine of the ten items loaded onto their hypothesized factors. The exception was Item 5 (‘I am not really interested in finding out the secret to a magic trick’), which was hypothesized to load on the Effect factor but instead loaded negatively on the Method factor (−.634). This suggests that a lack of interest in secrets is more closely related to low method orientation than to high effect orientation. Conceptually, this makes sense: disinterest in figuring out how a trick works reflects an absence of analytical engagement with magic rather than a positive investment in experiencing wonder. The item was subsequently reverse-scored and retained as part of the Method subscale. Overall, the exploratory factor analysis model was significant *χ*^2^(26, *N* = 922) = 177.51, *p* < .001, and both components had an eigenvalue greater than 1 (Method component eigenvalue of *l* = 3.700: Effect component eigenvalue of *l* = 1.484). Thus, the two-factor structure was retained, and no questions were removed from the scale.

**Table 2:** Results of exploratory factor analysis. Loadings of each MES item on the two factors.

**Item**	**Factor 1 (method)**	**Factor 2** ** (effect)**
Item 6	0.816	0.042
Item 10	0.796	−0.007
Item 7	0.741	−0.084
Item 9	0.653	0.021
Item 5	−0.634	0.115
Item 8	0.629	0.207
Item 1	0.147	0.730
Item 3	−0.130	0.664
Item 2	0.117	0.789
Item 4	−0.062	0.561

Next, a reliability analysis was conducted to assess the internal consistency of the factors (overall Cronbach’s Alpha = .83: method Cronbach’s Alpha = .86: effect Cronbach’s Alpha = .77). A paired samples *t*-test was conducted to compare mean scores on the Method and Effect subscales. Results indicated that participants scored significantly higher on the Method subscale than the Effect subscale (Method *M* = 3.62, *SD* = .81; Effect *M* = 3.52, *SD* = .75; *t*(921) = −3.13, *p* = .002), suggesting a slight overall preference for method orientation. However, as noted above, the two subscales are designed to measure independent dimensions.

### Method-Effect Scale correlations

Tables 3 and 4 present the descriptive statistics and correlations for the MES, ES and the EUB, the LOLS, the REI-10, and the CFW.

**Table 3:** Descriptive statistics.

Variables	*N*	*M*	*SD*
MES total	922	3.48	.54
MES effect	922	3.52	.75
MES method	922	3.62	.81
ES total	922	2.05	.61
EUB total	922	2.45	.52
EUB science knowledge	922	3.52	.42
EUB paranormal	922	2.14	.71
EUB religion	922	2.52	.76
EUB health	922	2.97	.48
EUB extraordinary	922	2.27	.73
EUB conspiracy theories	922	2.16	.56
EUB ghosts	922	2.65	.78
CFWS total	922	3.66	.81
CFWS perspective shifting	922	3.75	.96
CFWS emotional reawakening	922	3.56	.92
LOLS total	922	2.14	.76
REI total	922	3.46	.46
REI need for cognition	922	3.27	.63
REI faith in intuition	922	3.64	.63

**Notes.**

MES Method-Effect Scale SE Engagement Survey EUB Inventory of Epistemically Unwarranted Beliefs CFWS Capacity for Wonder Scale LOLS Loathing of the Legerdemain Scale REI Rational-Experiential Inventory-10

**Table 4:** Correlations between variables.

Variable	1	2	3	4	5	6	7	8	9	10	11	12	13	14	15	16	17	18	19
1. MES-T	–																		
2. MES-E	.78^***^	–																	
3. MES-M	.75^***^	.29^***^	–																
4. ES-T	.48^***^	.47^***^	.33^***^	–															
5. EUB-T	.00	.03	−.06	−.05	–														
6. EUB-P	.04	.06	−.02	.02	.87^***^	–													
7. EUB-R	−.04	−.02	−.07^*^	−.09^**^	.76^***^	.55^***^	–												
8. EUB-H	.01	.05	−.07^*^	−.05	.65^***^	.45^***^	.41^***^	–											
9. EUB-X	.01	.05	−.06	−.02	.82^***^	.65^***^	.49^***^	.42^***^	–										
10. EUB-C	−.14^***^	−.13^***^	−.13^***^	−.14^***^	.72^***^	.55^***^	.54^***^	.37^***^	.59^***^	–									
11. EUB-G	.08^*^	.07^*^	.04	−.01	.80^***^	.68^***^	.58^***^	.46^***^	.58^***^	.42^***^	–								
12. EUB-SK	.22^***^	.20^***^	.17^***^	.15^***^	−.22^***^	−.13^***^	−.36^***^	−.05	−.10^**^	−.39^***^	−.03	–							
13. CFWS-T	.34^***^	.26^***^	.27^***^	.29^***^	−.00	.01	−.03	.05	−.00	−.11^***^	.07^*^	.20^***^	–						
14. CFWS-PS	.27^***^	.15^***^	.25^***^	.22^***^	−.08^*^	−.05	−.10^**^	−.03	−.07^*^	−.16^***^	.04	.21^***^	.88^***^	–					
15. CFWS-ER	.33^***^	.30^***^	.22^***^	.28^***^	.08^*^	.07^*^	.04	.11^**^	.07^*^	−.03	.09^**^	.13^***^	.86^***^	.51^***^	–				
16. LOLS-T	−.53^***^	−.57^***^	−.36^***^	−.49^***^	.13^***^	.10^**^	.15^***^	.07^*^	.06	.21^***^	.03	−.26^***^	−.21^***^	−.13^***^	−.23^***^	–			
17. REI-T	.17^***^	.06	.20^***^	.07^*^	−.09^**^	−.07^*^	−.11^***^	−.00	−.10^**^	−.17^***^	.03	.17^***^	.37^***^	.42^***^	.22^***^	−.12^***^	–		
18. REI-NFC	.09^**^	.02	.15^***^	.06	−.20^***^	−.17^***^	−.18^***^	−.15^***^	−.16^***^	−.20^***^	−.09^**^	.09^**^	.26^***^	.33^***^	.12^***^	−.09^**^	.73^***^	–	
19. REI-FI	.15^***^	.06	.15^***^	.04	.08^*^	.07^*^	.03	.15^***^	.01	.05	.13^***^	.16^***^	.27^***^	.28^***^	.20^***^	−.08^**^	.73^***^	.06	–

**Notes.**

* *p* < .05.

** *p* < .01.

*** *p* < .001.

MES Method-Effect Scale ES Engagement Survey EUB Inventory of Epistemically Unwarranted Beliefs CFWS Capacity for Wonder Scale LOLS Loathing of the Legerdemain Scale REI Rational-Experiential Inventory-10

Variable abbreviations T Total E Effect M Method P Paranormal R Religion H Health X Extraordinary C Conspiracy Theories G Ghosts SK Science Knowledge PS Perspective Shifting ER Emotional Reawakening NFC Need for Cognition FI Faith in Intuition

### Engagement Survey

Both the MES Method (*r* = .33, *p* < .001) and the MES Effect (*r* = .47, *p* < .001) were positively associated with the ES scale.

### Loathing of Legerdemain Scale and Capacity for Wonder Scale

As expected, the MES Method and Effect subscales correlated negatively with the LOLS (Method *r* = −.36, *p* < .001; Effect *r* = −.57, *p* < .001) and positively with the CFW total scale and subscales (MES Method; Total *r* = .27, *p* < .001: Perspective shifting *r* = .25, *p* < .001: Emotional reawakening *r* = .22, *p* < .001: MES Effect; Total *r* = .26, *p* < .001; Perspective shifting *r* = .15, *p* < .001: Emotional reawakening *r* = .30, *p* < .001).

### Rational–Experiential Inventory-10

The MES Method subscale positively correlated with the REI-10 Need for Cognition subscale (*r* = .15, *p* < .001) and, contrary to predictions, was also positively correlated with the REI-10 Faith in Intuition subscale (*r* = .15, *p* < .001). However, both correlations are small in magnitude, and the Need for Cognition subscale demonstrated substandard internal consistency (*α* =.66) in this sample, so these associations should be interpreted cautiously. The MES Effect subscale was positively associated with the REI-10 Faith in Intuition subscale, although the correlation was small and non-significant (*r* = .06, *p* = .072).

### Inventory of Epistemically Unwarranted Beliefs

The MES total and the EUB total scale were not significantly associated (*r* = .00, *p* = 1.00). The MES Effect subscale positively correlated with the EUB scientific knowledge subscale (*r* = .20, *p* < .001) while showing mixed associations with other EUB pseudoscientific beliefs subscales. The MES Method subscale correlated positively with the EUB scientific knowledge subscale (*r* = .17, *p* < .001) and negatively with the EUB pseudoscientific beliefs subscales.

There were also associations between the MES subscales and specific EUB subscales. The MES Effect subscale was negatively associated with the EUB Conspiracy Theories subscale (*r* = −.13, *p* < .001) and positively associated with the EUB Ghosts subscale (*r* =.07, *p* = .038). The MES Method subscale was negatively associated with the EUB Conspiracy Theories (*r* = −.13, *p* < .001), Religion (*r* = −.07, *p* = .034), and Health (*r* = −.07, *p* = .028) subscales. However, several of these correlations are small in magnitude, and given the large sample size and the number of comparisons conducted, they should be interpreted with caution.

## Discussion

This study first developed a novel measure that examines whether individuals express a preference for the two main components involved in a magic trick: The effect and the method (the MES). As expected, the MES had a two-factor structure, comprising a Method and an Effect factor. The Method factor and its resulting items are related to an individual’s interest in magic, being attributed to their desire to understand the underlying mechanics of magic tricks. In contrast, the Effect factor and its items were related to an individual’s interest in magic, being attributed to their desire to feel awe and wonder rather than to solve the mechanism behind the magic trick. The data suggests that Method and Effect orientations are best understood as independent dimensions rather than competing preferences. In short, this study provides preliminary evidence that the MES is a psychometrically valid measure of interest in the Method and Effect components, as it demonstrates strong internal reliability and a factor structure consistent with the *a priori* model developed by the authors.

Evidence for convergent validity was established by its negative association with the LOLS, its positive association with the CFW overall and sub-scales (both perspective-shifting and emotional reawakening). Interestingly, the method and effect subscales of the MES did not load onto the REI-10 Need for Cognition and Faith in Intuition sub-scales as expected. Future studies should attempt to establish test-retest reliability and discriminant validity of the MES by evaluating concepts, such as the Big Five personality traits, as they were measured in prior research (Silvia et al., 2023).

The positive and significant relationships between the MES and Engagement Survey show that people’s interest in both Method and Effect is related to the degree to which they watch magic. However, the relationship between engagement and Effect was stronger than engagement and Method, suggesting that the former is a better predictor than the latter.

When assessing the relationships between the MES and the LOLS, both the method and Effect factors were negatively associated with the LOLS, as predicted. It was also found, as predicted, that the MES effect and Method components were positively associated with the ES questionnaire, as individuals more interested in the processes and experience of magic seem to interact more with magic in different capacities. We also found that the Method and Effect subscales positively correlated with the CFW and its emotional reawakening and perspective-shifting subscales. For the MES and its associations with the REI, the Method factor was positively correlated with both the REI Faith in Intuition and Need for Cognition subscales, while the Effect factor was only positively associated with the REI Faith in Intuition scale. The exploratory analysis of the relationship between the MES and the EUB yielded a complex pattern. Both the Method and Effect subscales were positively associated with the EUB Scientific Knowledge subscale, suggesting that interest in magic, regardless of orientation, is associated with greater scientific literacy. The remaining associations were small in magnitude and inconsistent across subscales, providing little evidence that either orientation is broadly linked to endorsement of epistemically unwarranted beliefs. However, it is possible that these small correlations hide a more complex picture. For example, people scoring high on the MES Effect subscale might be a mixture of those that believe in the paranormal and those who simply enjoy seeing an apparently impossible event. Future research could examine whether this is the case.

The scientific investigation of the reasons why individuals are interested in magic is a relatively new area of research. Previous research conducted by Silvia et al. (2025a) found that there are two distinct groups of individuals who like magic: a group high in critical thinking and another group that scores highly in belief in paranormal phenomena. Our study identifies a further distinction within people’s appreciation of magic, showing that interest in the method and interest in the effect are best understood as related but distinct dimensions rather than as mutually exclusive orientations. Rather than sorting spectators into separate groups, the MES allows a given individual to score high or low on either or both dimensions, and the positive correlation between the two subscales (*r* = .29) indicates that valuing one component does not preclude valuing the other. Like Silvia et al. (2023), our results indicate that interest in both components of magic is associated with greater openness to experiences that challenge our preconceived notions about the world (perspective-shifting) and to events that elicit epistemic emotions (emotional reawakening).

Our exploratory analysis found limited evidence that preference for either the Method or Effect components of magic is broadly associated with epistemically unwarranted beliefs. This is noteworthy given that prior literature suggests interest in magic is linked to the belief that magical performances involve supernatural abilities (Silvia et al., 2025b), and that such beliefs are in turn associated with belief in psychic powers and precognition. One possible explanation is that the MES captures interest in the craft and experience of magic rather than beliefs about its nature. That is, someone who scores high on the Effect subscale may thoroughly enjoy the feeling of impossibility without attributing it to genuine supernatural forces. Future studies should investigate this distinction by pairing the MES with measures of magical thinking and supernatural attribution to determine whether interest in magic and belief in magic are separable constructs.

The MES was conceived around the notion that magic tricks can be seen as puzzles to be solved (method oriented) or impossible events that elicit a sense of wonder and astonishment (effect oriented). However, one referee rightly noted that some magic theorists have argued that a genuine sense of wonder (within a magic show) comes from discounting all possible methods (*e.g.*, Aronson, 1990; Ortiz, 1995; Leddington, 2016; Lamont, 2017). If this is the case, high scores on the MES Method scale might be related to a certain type of wonder (*e.g.*, a more intellectually based experience in which it isn’t possible to figure out how a trick is achieved), whilst high scores on the MES Effect scale might be associated with another type of wonder (*e.g.*, a more emotional experience related to concepts such as beauty and escapism). Future work could investigate this possibility, perhaps by carrying out more qualitative examination of how those with different MES profiles perceive and experience magic.

The MES also has the potential to be applied in other domains. For instance, other researchers suggest that magic-related educational programs could be used to improve well-being (Bagienski & Kuhn, 2019). Particularly, the authors suggest that using magic as a teaching mechanism has the most potent effect. Much research has also focused on how magic interventions can be used to improve various psychological emotions and well-being. For example, having individuals perform magic to improve self-efficacy (Bagienski et al., 2022), using magic to improve self-esteem and depression in older adults (Wang et al., 2023), and using magic to improve neurocognitive performance in older adults (Lee et al., 2022). Thus, the MES may be used as a pre-screening tool to better understand which aspect of magic (Method *versus* Effect) individuals align with most to enhance their experience when learning with magic, particularly magic-related educational and wellbeing programs.

The MES also presents the opportunity to gain a more robust understanding of how individuals interact with magic tricks from a psychological perspective. Lan et al. (2018) found that magical demonstrations can be used to influence individuals’ belief that magic is real, regardless of whether the demonstrator was described as a magician or a psychologist. Given that the MES is a measure of magic interest, it could be employed to assess how interest in the Method or Effect component of magic shapes how individuals’ beliefs are influenced by magical demonstrations. More broadly, magicians could use the MES to better understand which component the audience connects a magic trick with. This decomposition of magic into method and effect components may enhance our current understanding of the cognitive mechanisms involved in the experience of magic (Cole & Kuhn, 2025; Rensink & Kuhn, 2015). For instance, some authors suggest that an individual’s reaction to the seemingly impossible happenings during a magic trick elicits a sense of cognitive dissonance (Leddington, 2016). Since the MES measures interest in both components of magic tricks, it could be used in future research to better understand how interest in a particular component of magic influences the degree of cognitive dissonance magic tricks generate.

Magic tricks and their associated performance are also heavily influenced by ecological context. For instance, a magic trick performed live in front of other participants may be perceived as vastly different compared to a trick viewed in video format. Prior research has shown that individuals prefer magic when viewed live rather than in a video format (Kuhn et al., 2025). Other environmental factors that can influence the experience of magic include the proximity to the magician (Leddington, 2016) and the relationship between the magician and the spectator (Pailhès & Kuhn, 2020). Therefore, future research using the MES could experimentally manipulate the environment in which magic is performed to further probe how interest in both the method and effect components of magic is changed by ecological factors. This would enable magicians to refine their tricks and performances for both entertainment and educational purposes. Moreover, future research into MES variations could include integrating different factors that impact magic engagement, such as modified questions from the ES.

### Limitations

Despite the novel implementation of the MES as a measure of interest in the method and effect components of magic tricks and the subsequent exploration of the scale’s correlates, the study has several limitations. Firstly, the population for the current study was comprised of a large undergraduate student sample. Past research has shown that the educational and well-being benefits of magic are often beneficial for children and older adults (Kovács, 2023; Lee et al., 2022; Lee et al., 2024; Wiseman & Kaye, 2020; Wiseman & Watt, 2020). Therefore, including children and older adults may have revealed population differences when using the MES, limiting the generalizability of the data collected.

Another potential limitation of the study is the exclusion of other relevant constructs of interest in magic tricks that would have enabled a further exploration of the validity of the MES. For instance, Silvia et al. (2025b) found that individuals who had a higher level of interest in magic were more likely to suggest that a magician’s tricks were due to paranormal abilities. This suggests that an inclusion of scales measuring concepts such as magical thinking (*i.e.,* an individual’s capacity to think about magical concepts) and magical beliefs (*i.e.,* belief that magic can exist in reality) (Subbotsky, 2014) may have further aided in adapting the MES to better inform how the scale could measure differential interest in the method and effect components of magic tricks. Moreover, prior studies measuring liking of magic also included measures of personality, such as the Big Five Personality Traits, intolerance of uncertainty and the dark and light triads of personality (Silvia et al., 2023), which would have allowed for more robust convergent validity. As well, the questionnaires were presented in a fixed order rather than being randomized or counterbalanced. Although the MES was presented first to ensure that the other measures did not influence responses to the new scale, responses to the later questionnaires may have been subject to order effects, including fatigue. Future research could counterbalance the order of the measures to address this issue.

Lastly, while participants were asked about how they consume and interact with magic, they were not asked about how different ecological variables impact the degree to which they are interested in magic. Asking participants questions about these contextual factors, such as whether they prefer live in-person magic or audiovisual magic, may have provided additional insights into how ecological circumstances influence interest in method and effect components of magic. This could also be extended to what types of magic individuals may be interested in. For instance, matter manipulation could be more engaging for those interested in the effect component of magic, whereas mentalism could be more engaging for those intrigued by the method component. Thus, correlating different ecological variables may have provided meaningful context for specific research where the MES could be used.

## Conclusions

As scientific exploration of the benefits of magic for wellbeing and education purposes grows, finding ways to hone our understanding of why individuals engage with magic is critical. The current study details the creation of the MES, a novel scale for measuring interest in the method and effect components of magic tricks. We found evidence that the MES is a short, valid two-factor measure that could be used in various contexts to enhance our understanding of why individuals are interested in magic. We also found robust evidence indicating that the MES method and effect components correlate to convergent variables, such as rational and intuitive thinking, epistemic emotions such as wonder, and dislike of magic. Moving forward, research should assess how ecological context can influence the MES, further examine the validity and reliability of the MES and further evaluate how the MES may be used as a pre-screening tool to increase the efficacy of magic-related well-being and education programs. While examining magic scientifically is a relatively new area of research, tools that assess how magic can be harnessed for various beneficial programs provide an opportunity to demonstrate that magic entertainment has depth behind its tricks.

##  Supplemental Information

10.7717/peerj.21742/supp-1 Supplemental Information 1 Raw data AGE–Age: GEN–Gender (1 = male, 2 = female, 3 = gender non-conforming, 4 = preferred not to respond): ATNC—Attention test EUBTOT—EUB Total: EUBPARA—EUB Paranormal Subscale EUBREL—EUB Religion Subscale: EUBHEA—EUB Health Subscale EUBELF—EUB Extraordinary Life Forms Subscale: EUBCONT—EUB Conspiracy Theories Subscale: EUBGH—EUB Ghosts Subscale EUBSK—EUB Science Knowledge Subscale: REINFC—REI Need For Cognition Subscale: REIFI—REI Faith in Intuition: REITOT—REI Total CFWTOT—Capacity For Wonder Total: MES-E—Method Effect Effect Subscale: MES-M—Method Effect Method Subscale: ESTOT—Engagement Survey Total: CFWPS—Capacity for Wonder Perspective Shifting Subscale: CFWER—Capacity for Wonder Emotional Reawakening Subscale: LOLSTOT—Loathing of Legerdemain Total MESTOT—Method Effect Total

10.7717/peerj.21742/supp-2 Supplemental Information 2 File showing how the categorical data was coded Each of the codings used for every scale

## Appendix A: Engagement survey

1. **How often do you watch magic performances online or on television?**

*(1*=* Never, 2*=* Less than once a month, 3*=* 1–3 times a month, 4*=* Once a week, 5*=* Multiple times a week)*

2. **How often do you attend live magic performances?**

*(1*=* Never, 2*=* Once a year or less, 3*=* 2–3 times a year, 4*=* 4–6 times a year, 5*=* More than 6 times a year)*

3. **How much do you enjoy watching magic performances online or on television?**

*(1*=* Not at all, 2*=* A little, 3*=* Somewhat, 4*=* A lot, 5*=* Very much)*

4. **How much do you enjoy attending live magic performances?**

*(1*=* Not at all, 2*=* A little, 3*=* Somewhat, 4*=* A lot, 5*=* Very much)*

5. **Approximately how many live magic shows have you attended in your life?**

*(1*=* None, 2*=* 1–2 shows, 3*=* 3–5 shows, 4*=* 6–10 shows, 5*=* More than 10 shows)*.

## Additional Information and Declarations

### Data Availability

The following information was supplied regarding data availability: The data is available in the Supplemental Files.
