# Supplementary material for: A magical experience or a puzzle: how do people watch conjuring tricks?
Source: PeerJ. 2026 Sep 25;14:e21742. doi: 10.7717/peerj.21742 (PMC13618447; doi:10.7717/peerj.21742)
Supplement: Supplementary file 1 [file peerj-14-21742-s002.docx]

**DATA CODEBOOK**

The categorical data was numerically recorded in the following ways:

**GEN**
Gender coded in the following way
1: Male

2: Female
3: Gender non-conforming
4: Prefer not to respond

**ATNC**
Attention test (If you are reading this, select option "c." ), coded:

1: a

2: b

3: c

4: d

5: e

**Method-Effect Scale (MES)**

1 - Strongly disagree

2 - Disagree

3 - Neutral

4 - Agree

5 - Strongly agree

**Inventory of Epistemically Unwarranted Beliefs**

1 - Sure it's false

2 - Uncertain, but think it's false

3 - Really don't know

4 - Uncertain, but think it's true

5 - Sure it's true

**Loathing of Legerdemain Scale**

1 - Strongly disagree

2 - Disagree

3 - Neutral

4 - Agree

5 - Strongly agree

**Capacity for Wonder Scale**

1 - Not at all likely

2 - Slightly likely

3 - Somewhat likely

4 - Moderately likely

5 - Very likely

6 - Extremely likely

**Rational–Experiential Inventory–10**

1 - Completely false

2 - Mostly false

3 - Neutral

4 - Mostly true

5 - Completely true

**Engagement Survey**

**How often do you watch magic performances online or on television?**
1 - Never

2 - Less than once a month

3 - 1-3 times a month

4 - Once a week

5 - Multiple times a week

How often do you attend live magic performances?

1 - Never

2 - Once a year or less

3 - 2-3 times a year

4 - 4-6 times a year

5 - More than 6 times a year

How much do you enjoy watching magic performances online or on television?

1 - Not at all

2 - A little

3 - Somewhat

4 - A lot

5 - Very Much

How much do you enjoy attending live magic performances?

1 - Not at all

2 - A little

3 - Somewhat

4 - A lot

5 - Very Much

Approximately how many live magic shows have you attended in your life?

1 - None

2 - 1-2 shows

3 - 3-5 shows

4 - 6-10 shows

5 - More than 10 shows
